# 443. Albumin is a Reliable and Accurate Biomarker for Assessing Clinical Course of Multisystem Inflammatory Syndrome in Children

**DOI:** 10.1093/ofid/ofad500.513

**Published:** 2023-11-27

**Authors:** Bazak Sharon, Conor Nath

**Affiliations:** University of Minnesota, Minneapolis, Minnesota; University of Washington, Minneapolis, Minnesota

## Abstract

**Background:**

In Spring 2020, Multisystem Inflammatory Syndrome in Children (MIS-C) emerged as a life-threatening pediatric complication to COVID-19. It is a complex and novel disease often requiring high levels of intensive care. As case definitions and guidelines are still being developed, it is imperative to identify biomarkers that can accurately assess its clinical course in hospitalized children.

**Methods:**

Utilizing the electronic medical records we retrieved the charts of children who were hospitalized at the University of Minnesota Masonic children’s Hospital for treatment of MIS-C. We reviewed their clinical course including daily monitoring of various inflammatory markers and analyzed the trends of albumin and CRP.

**Results:**

Between May 2020 and January 2022, thirty-six children diagnosed with MIS-C were admitted to the University of Minnesota Masonic children’s Hospital [Figure 1]. Complications included myocarditis, coronary aneurysm, coagulopathy, acute kidney injury, and shock. Seventeen children required PICU stay. All patients had elevated CRP on presentation, and values typically started to decline within 24-48 hours [Figure 2]. However, hypoalbuminemia continued to worsen for 4-5 days after admission, especially for patients requiring intensive care [Figure 3]. Both markers gradually improved for the entire cohort, reflecting the positive outcome our patients demonstrated.

**Figure:**
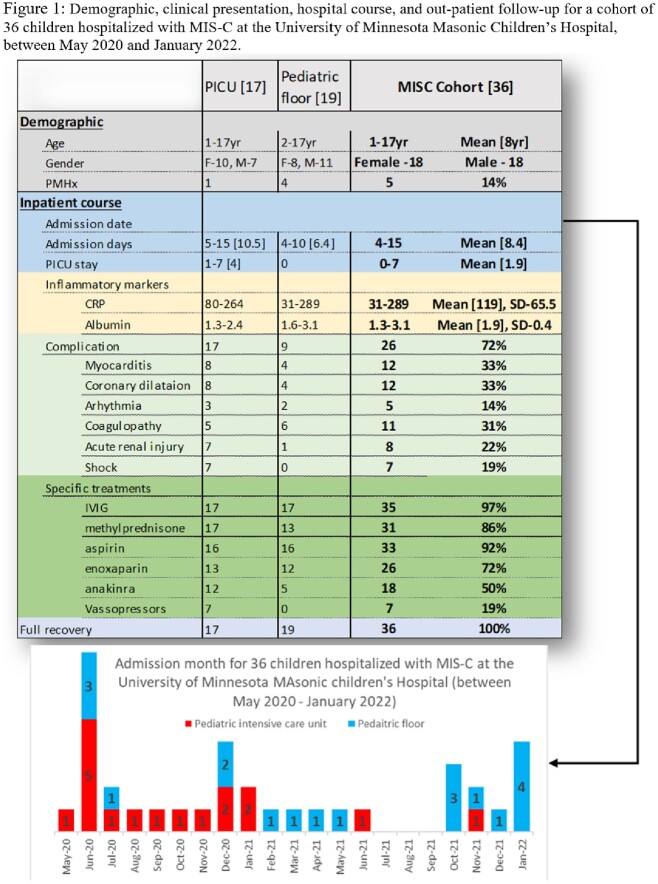

**Figure:**
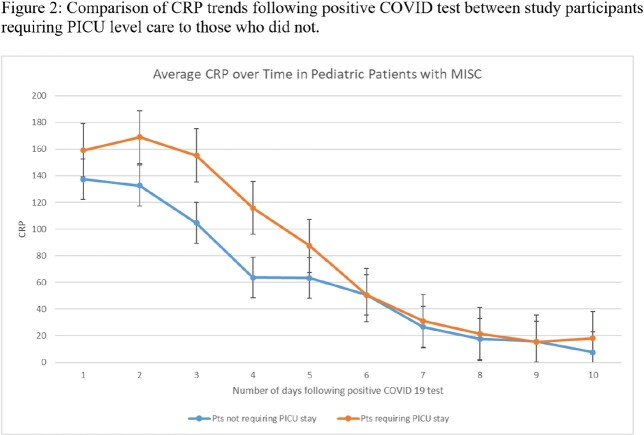

**Figure:**
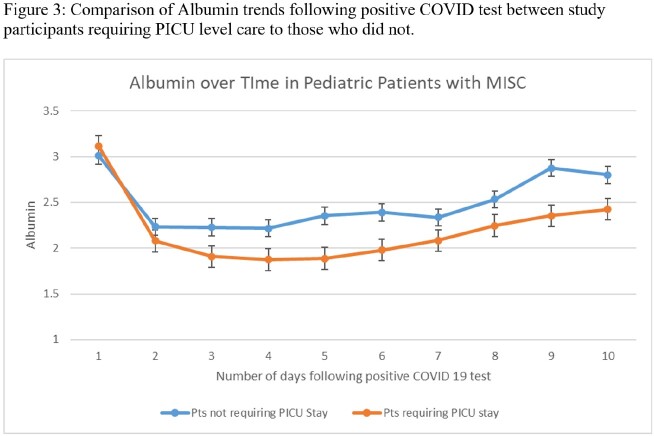

**Conclusion:**

Our study highlights the reliability of CRP and albumin as biomarkers for assessing MIS-C patients. While CRP values showed a wide range and high variability throughout the course of illness, albumin appeared to be more accurate, particularly in critically ill children. We recommend regular monitoring of albumin as an inflammatory marker and suggest frequent follow-up for children with systemic inflammatory disease.

**Disclosures:**

**All Authors**: No reported disclosures

